# Decision-making structure and nudges for Japanese pig farmers to implement biosecurity measures against classical swine fever outbreaks

**DOI:** 10.3389/fvets.2026.1865451

**Published:** 2026-06-24

**Authors:** Makoto Ukita, Yujin Katada, Kohei Makita

**Affiliations:** Veterinary Epidemiology Unit, School of Veterinary Medicine, Rakuno Gakuen University, Ebetsu, Japan

**Keywords:** behavioral economics, biosecurity measure, classical swine fever, CSF, decision-making, KAP, nudges, protection motivation theory

## Abstract

**Introduction:**

This study evaluated the decision-making structure that influence pig farmers to implement biosecurity measures to prevent classical swine fever (CSF) outbreaks and identified sociological factors affecting implementation in order to explore effective intervention strategies.

**Methods:**

A cross-sectional questionnaire survey was conducted among pig farmers in Japan to collect data on farm and farmer characteristics, the implementation of biosecurity measures, and farmers’ awareness of and motivation to carry out such measures. Questionnaires were distributed to 516 farms across 15 prefectures, and the analysis was conducted using data from 228 farms with valid responses. Structural equation modeling was applied to quantitatively analyze the decision-making process based on the knowledge, attitude, practice, and capacity (KAP-C) and the protection motivation theory (PMT) frameworks. In addition, relationships between farm characteristics and biosecurity measure implementation, as well as factors derived from nudge theory, were evaluated.

**Results:**

As a result, the latent variable Capacity was not identified in the KAP-C model, and Knowledge was associated with Attitudes (*β* = 0.99), which was also with Practice (*β* = 0.83). Similarly, in the PMT model, Threat (*β* = 0.43) and Coping (*β* = 0.56) appraisals were associated with Action. In both models, risk and threat perceptions were associated with Practice. In contrast, farms with higher capacity, such as large-scale farms (*p* < 0.001) and those managed by younger owners (*p* = 0.050), had higher rates of biosecurity implementation. Farms with a history of CSF outbreaks also demonstrated higher implementation rates (*p* < 0.001). Guidance from veterinary clinicians (*ρ*= 0.24, *p* < 0.001) and Livestock Hygiene Service Centers (LHSCs; *ρ* = 0.16, *p* = 0.014), integrating measures into routine farm work positively affected biosecurity practices, and commitment such as goal setting (*ρ* = 0.46, *p* < 0.001) and community-based engagement (*ρ* = 0.35, *p* < 0.001) positively influenced the implementation of biosecurity measures.

**Discussion:**

Farmers may respond effectively to messages in which veterinary clinicians and LHSCs collaborate to clearly present effective biosecurity measures and disease threats, foster concern for pigs, and encourage participation in biosecurity practices at the community level.

## 1 Introduction

The implementation of biosecurity measures on pig farms is critically important for preventing infectious diseases such as classical swine fever (CSF) and African swine fever (ASF), which cause severe economic losses not only on affected farms but also at the regional and national levels. In Japan, CSF re-emerged on pig farms in September 2018 after an absence of 26 years (1, 2), and despite the subsequent introduction of a nationwide CSF vaccination program (3), sporadic outbreaks have continued as of February 2026. Effective prevention and control of CSF or ASF require the thorough implementation of farm-level biosecurity measures, including control of vehicle and human movements, prevention of wildlife intrusion, and proper hygiene management within pig houses (4, 5). However, implementation of these measures depends largely on the routine and habitual behaviors of farm owners and workers, and substantial variation in implementation levels among farms has been reported (3, 4).

Previous studies have shown that farm characteristics such as size and management type affect the implementation of biosecurity measures (6). However, these characteristics alone may not fully explain variations in implementation. In addition to structural factors such as resources and facilities, psychological and social factors related to decision making can influence the adoption of biosecurity measures. Focusing on these factors is important for obtaining a deeper understanding of biosecurity practices and for designing effective intervention strategies.

Sociological frameworks have been widely used to evaluate barriers to infectious disease control on livestock farms. In particular, the Knowledge, Attitudes, and Practices (KAP) framework is commonly applied (7). An extension of the KAP model that incorporates the concept of Capacity, known as KAP-C (KAP and Capacity), allows for analyses that consider how farmers’ abilities and resources influence decision making (8). In addition, social psychological approaches have been applied to examine the decision-making structure of pig farmers with regard to adopting biosecurity measures (9, 10). Protection Motivation Theory (PMT) proposes that two cognitive processes, Threat appraisal and Coping appraisal, determine the execution of protective behaviors (11). In Threat appraisal, recognition of infection risks and disease severity serves as a motivator for action, whereas in Coping appraisal, beliefs concerning the effectiveness of measures (known as response efficacy and self-efficacy) enhance the willingness to implement such measures. These theoretical models are useful for understanding how farmers’ knowledge, attitudes, threat perception, and capacity affect the implementation of biosecurity measures.

Nudge theory is a concept in behavioral economics designed to guide individuals toward engaging in desirable behaviors while preserving their freedom of choice (12). This theory is also reportedly useful in the field of public health when applied to promote healthy behaviors and increase uptake of preventive interventions such as vaccination (13). MINDSPACE, developed by the Institute for Government and Behavioral Insights Team in the United Kingdom, is a concrete framework for implementing nudge interventions (14). MINDSPACE incorporates nine psychological mechanisms that influence human behavior, namely Messenger, Incentives, Norms, Defaults, Salience, Priming, Affect, Commitments, and Ego. The name of the framework was derived from the first letters of the nudge component terms. Utilizing these mechanisms enables the design of interventions that encourage farmers to engage in desired behaviors without coercion. Strategies to enhance farmer compliance with animal disease control measures in the context of nudge theory have been examined in Europe (15), and in Vietnam, nudge strategies have been explored as a means of improving food safety management practices along the pork value chain (16). However, the application and evaluation of nudge theory in the context of implementing biosecurity practices on Japanese pig farms remain limited.

The aim of this study was to clarify the decision-making structure among Japanese pig farmers with regard to the adoption of biosecurity measures to prevent CSF outbreaks, based on the KAP-C and PMT frameworks. Furthermore, the study investigated sociological factors and nudges that affect implementation in order to explore interventions that motivate pig farmers to voluntarily implement these measures.

## 2 Materials and methods

### 2.1 Study design and population

**Figure 1:**
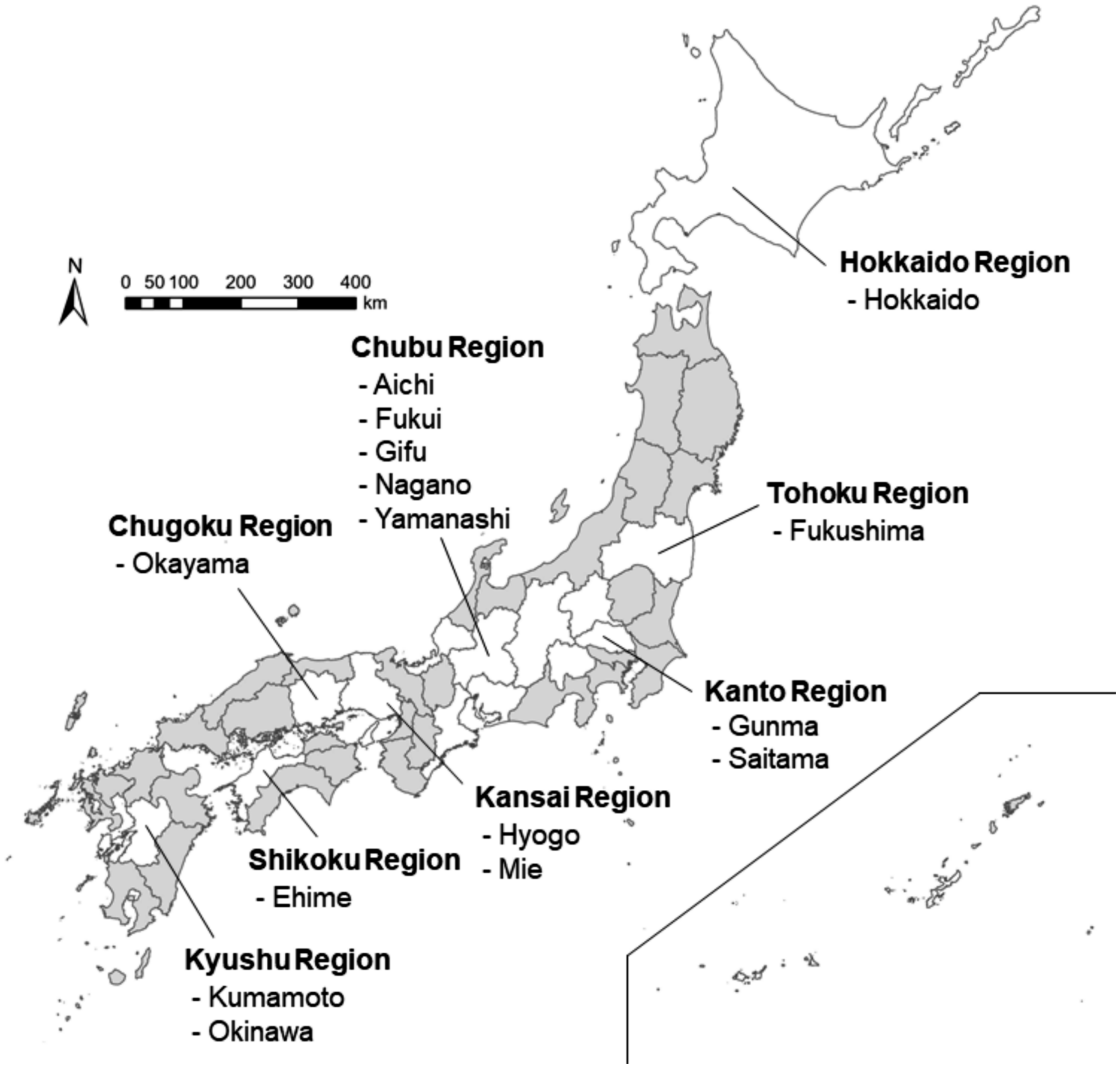
Prefectures included in the questionnaire survey conducted between May and October 2021. White areas indicate the 15 prefectures surveyed across Japan.

A cross-sectional postal survey using a structured questionnaire was conducted to investigate the implementation of biosecurity measures against CSF on pig farms as well as farmers’ perceptions and motivations regarding biosecurity. The survey targeted 516 pig farms located in 15 prefectures across Japan, with at least one prefecture selected from each region (Figure 1). The prefectures included those selected in consultation with the Ministry of Agriculture, Forestry, and Fisheries (MAFF) and relevant prefectural authorities from each region, as well as the 4 regions comprising 9 prefectures where CSF outbreaks were reported between September 2018 and October 2019 (Aichi, Fukui, Gifu, Gunma, Mie, Nagano, Okinawa, Saitama, and Yamanashi).

The target population consisted of farmers managing pig farms of various production types, including breeding, fattening, and farrow-to-finish. This study was conducted in parallel with another published study aimed at identifying effective biosecurity measures for CSF prevention in regions affected by CSF outbreaks between September 2018 and October 2019 (4). Accordingly, in the nine prefectures where CSF outbreaks were reported during that period, all CSF outbreak farms and farms located within a 10-km radius of these outbreak farms were included in the study. In the other prefectures, farmers from 70 randomly selected farms were invited to participate if the number of pig farms exceeded 70, whereas all farms were included when fewer than 70 pig farms were present.

The present study employed the structural equation modeling (SEM) in clarifying decision-making structure for the implementation of biosecurity measures, and the calculation of sample size *N* in the SEM requires effect size γ , discrepancy function $F_{0}$ , and degree of freedom d (Equations 1, 2) (17).


$$\varepsilon =\sqrt{\frac{F_{0}}{d}}$$ (1)

$$\gamma =(N-1)d\varepsilon^{2}$$ (2)

where ε is the root mean square error of approximation (RMSEA). At the planning phase, the structure of SEM or exact number of variables in a questionnaire used in the SEM, which are required for calculating d , and γ itself cannot be determined. Therefore, given the survey was relatively large scale, the sample size was considered sufficient for the analysis.

Participation was voluntary, and respondents were assured of anonymity and confidentiality. The study protocol was approved by the Research Ethical Committee of Rakuno Gakuen University (approval number: 20-5), and completion of the questionnaire was considered implied consent.

### 2.2 Questionnaire design and data collection

A structured questionnaire was developed with the cooperation of four veterinarians, each with more than 5 years of experience in swine clinical practice. A pilot survey was conducted involving seven pig farmers who were not included in the main study, and the questionnaire was revised based on their feedback. The final questionnaire consisted of 10 sections covering: (i) farmers’ attributes and basic farm information, (ii) biosecurity practices, including preventing the (a) introduction of pathogens into the hygienic control area by farm staff, (b) introduction of pathogens into pig houses by farm staff, (c) spread of pathogens within pig houses, (d) introduction of pathogens onto the farm by external visitors, and (e) introduction of pathogens into pig houses by wildlife (Table 1), and (iii) farmers’ awareness of and motivation to implement farm biosecurity practices (Table 2). The biosecurity questionnaire items were developed based on epidemiological investigations during CSF outbreaks and advice from swine clinical veterinarians, rather than a standardized scoring system. Development of the survey and final questionnaire was based on the components of the MINDSPACE framework, KAP-C, and PMT.

**Table 1:** Questionnaire items related to farmers’ attributes, basic farm information, and biosecurity practices.

Category	Item
(i) Farmers’ attributes and basic farm information	Age; gender; business structure; production type; number of workers; number of sows and fatting pigs; CSF outbreak experience.
(ii) Biosecurity practices	
(a) Introduction of pathogens into the hygienic control area by farm staff (5 items)	Disinfecting farm staff vehicles; properly adjusting the concentration of disinfectant for vehicles; clarifying the border of the hygienic control area; conducting showering-in; changing into special clothing.
(b) Introduction of pathogens into pig houses by farm staff (5 items)	Daily washing and disinfecting of special clothing for each pig house; changing into special clothing at the entrance of each pig house; changing into special boots at the entrance of each pig house; establishing clear zones in which to place outer and inner boots; disinfecting hands or wearing hygienic gloves.
(c) Spread of pathogens within pig houses (5 items)	No moving of pigs by having them walk on the ground; adoption of all-in and all-out for pig pens; disinfection of the pens after an all-out; adoption of all-in and all-out for pig rooms; disinfection of the rooms after an all-out.
(d) Introduction of pathogens into the farm by external visitors (9 items)	Restricting entry of feed transport vehicle into the hygienic control area; disinfecting veterinarians’ vehicles; changing into special boots by veterinarians; cleaning veterinarians’ hands before providing veterinary services; disinfecting construction tools transported onto the farm; changing into special clothing by representatives of repair services; changing into special boots by facility construction services; disinfecting hands or wearing hygienic gloves by facility construction services; restricting entry of visitors without clear reason to be on the farm.
(e) Introduction of pathogens into pig houses by wildlife (5 items)	Preventing wildlife intrusion into areas used to store pig carcasses; preventing the intrusion of mice and other small wild animals into pig houses; preventing the intrusion of cats, raccoons, and other medium-sized wild animals into the hygienic control area; preventing the intrusion of feral cats, raccoons, and other medium-sized wild animals into pig houses; preventing the intrusion of wild birds into pig houses.

**Table 2:** Questionnaire items assessing pig farmers’ awareness and motivation, arranged according to MINDSPACE nudge* and the KAP-C** and PMT*** frameworks.

Item	Nudge	KAP-C	PMT
Incorporating advice from veterinarians	Messenger	K to A	–
Incorporating advice from LHSCs	Messenger	K to A	–
Incorporating advice from neighboring pig farmers	Messenger	K to A	–
Incorporating advice from animal pharmaceutical suppliers	Messenger	K to A	–
Understanding the impact of CSF or ASF outbreaks on farms	Incentives	K to A	Severity or vulnerability
Understanding the impact of CSF or ASF outbreaks on the region or country	Incentives	K to A	Severity or vulnerability
Concerns about inadequate hygiene management conditions on neighboring pig farms	Incentives and norms	K to A	Severity or vulnerability
Belief in the prevention of CSF intrusion onto the farm by vaccinating pigs against CSF	Incentives	K to A	Severity or vulnerability
Satisfaction with own farm biosecurity measures	Incentives and ego	K to A	Intrinsic rewards
Implementation of biosecurity measures in order to increase revenue	Incentives	K to A	Extrinsic rewards
Implementation of biosecurity measures through understanding the risk of diseases entering the farm	Incentives	K to A	Response or self-efficacy
Belief in the prevention of CSF and ASF intrusion onto the farm by preventing the intrusion of wild animals	Incentives	K to A	Response or self-efficacy
Belief in the prevention of CSF and ASF intrusion onto the farm by thorough disinfection of vehicles entering the farm	Incentives	K to A	Response or self-efficacy
Belief in the prevention of CSF and ASF intrusion onto the farm by implementing hygiene management for individuals entering the farm	Incentives	K to A	Response or self-efficacy
Belief in the prevention of CSF and ASF intrusion onto the farm by implementing feed and water hygiene management practices	Incentives	K to A	Response or self-efficacy
Belief in the prevention of CSF and ASF intrusion onto the farm by implementing hygiene management practices in pig houses	Incentives	K to A	Response or self-efficacy
Being mindful of one’s reputation among wholesalers and consumers	Norms	A	Extrinsic rewards
Being mindful of one’ reputation among neighboring pig farmers	Norms	A	Extrinsic rewards
Being mindful of one’ reputation among veterinarians	Norms	A	Extrinsic rewards
Being mindful of one’ reputation among LHSCs	Norms	A	Extrinsic rewards
Desire to maintain biosecurity at a level similar to neighboring pig farms	Norms	A	Extrinsic rewards
Belief that if effectiveness is clearly demonstrated, biosecurity measures can be implemented	Defaults	K	Response or self-efficacy
Understanding the significance of the biosecurity measures currently performed as part of the routine	Defaults	K	Response or self-efficacy
Belief that biosecurity measures can be implemented through routine practices	Defaults	K to A	Response or self-efficacy
Willingness to accept new practices	Defaults and salience	A	Response or self-efficacy
Educating farm staff regarding biosecurity practices	Salience	K to A	Intrinsic rewards or Severity or vulnerability
Participation in workshops on biosecurity measures for livestock infectious diseases	Salience	K to A	Intrinsic rewards or Severity or vulnerability
Conducting studies on farm Hazard Analysis Critical Control Point (HACCP) and Japan Good Agricultural Practices (J-GAP)	Salience	K to A	Intrinsic rewards or Severity or vulnerability
Engaging in a study of livestock hygiene information distributed by the LHSCs	Salience	K to A	Intrinsic rewards or Severity or vulnerability
Willingness to study the current CSF epidemic	Salience	K to A	Severity or vulnerability
Willingness to study ASF, for which there is potential risk of entry into Japan	Salience	K to A	Severity or vulnerability
Interest in branding that can promote the thorough implementation of biosecurity measures	Salience and ego	A	Intrinsic or extrinsic rewards
Consideration of hand sanitization on the farm due to the impact of COVID-19	Priming	A	Response or self-efficacy
Use of agriculture-related magazines as a reference for biosecurity practices	Priming	A	Response or self-efficacy
Desire to reduce diseases in pigs out of care and affection	Affect	K to A	Intrinsic or extrinsic rewards
Setting goals for practicing biosecurity	Commitments	A	Intrinsic rewards
Active collaboration with other farms to enhance hygiene levels in the region	Commitments	A	Extrinsic rewards
Belief that the current number of farm staff is sufficient	–	C	Self-efficacy
Belief that there are enough labor hours to implement current biosecurity measures	–	C	Self-efficacy
Belief that the budget is sufficient to implement current biosecurity measures	–	C	Response costs

*MINDSPACE, messenger, incentives, norms, defaults, salience, priming, affect, commitments, and ego.

**KAP-C, knowledge, attitude, practice, and capacity.

***PMT, protection motivation theory-intrinsic/extrinsic rewards, severity or vulnerability, response or self-efficacy, and response costs.

For questions regarding the actual implementation of biosecurity measures, respondents were asked to report only those measures currently in place at the time of the survey, considering that practices may have changed following CSF outbreaks. Most questions regarding biosecurity measures and farmers’ perceptions and motivations were answered using a five-point Likert scale (ranging from 1: never/very poor/strongly disagree to 5: always/excellent/strongly agree). Some questions required nominal, numerical, or yes/no responses.

Questionnaires were posted to the target pig farms via prefectural government offices or Livestock Hygiene Service Centers (LHSCs) in May 2021. In Hokkaido Prefecture, the questionnaires were distributed to member farmers by the Hokkaido Pig Producers Association. Completed questionnaires were returned to Rakuno Gakuen University by the end of October 2021. Responses from each farmer were reviewed, and questionnaires with responses to at least 70% of the items were considered valid.

Scores for questions related to biosecurity practices and farmers’ awareness and motivation were calculated as the mean score for each item. To assess nudges, items were classified according to the nine psychological mechanisms of the MINDSPACE framework, and scores within each group were compared using the Wilcoxon signed-rank test. For pairwise comparisons, *p* values were adjusted using the Bonferroni correction.

### 2.3 Structural equation modeling based on the frameworks designed for supporting the decision-making process

SEM (18) based on the KAP-C and PMT frameworks was conducted to quantitatively evaluate the decision-making process underlying the implementation of biosecurity measures by pig farmers. In the KAP-C framework, increased Knowledge was assumed to positively influence Attitude, which in turn would affect the decision to implement biosecurity measures, whereas higher Capacity facilitates Knowledge acquisition and measure implementation (8), leading to four latent variables: Knowledge, Attitude, Practice, and Capacity. In the PMT framework, protection motivation was assumed to be influenced by Threat appraisal and Coping appraisal. Threat appraisal was assumed to increase with the perceived severity and vulnerability of CSF and ASF outbreaks. Threat appraisal was also assumed to increase with the satisfaction and fulfillment (intrinsic rewards) and social approval (extrinsic rewards) derived from implementing biosecurity measures. By contrast, Coping appraisal was assumed to increase with the perceived effectiveness of measures (response efficacy) and self-efficacy but decrease with perceived burdens and costs. Based on these assumptions, three latent variables were defined: Threat appraisal, Coping appraisal, and Action.

Observed variables for SEM included biosecurity practice variables and farmers’ awareness and motivation variables. Missing values averaged 1.3% (range: 0–4.0%) for biosecurity variables and 2.5% (range: 0.4–7.5%) for awareness and motivation variables, and missing values were imputed using the random forest algorithm in the R package “missForest” (19). Representative variables for each biosecurity practice category were selected after examining correlations and distributions, avoiding variables with extremely skewed values. Candidate awareness and motivation variables were selected based on regression analyses using biosecurity variables as the outcome (*p* < 0.2); variables with similar meanings were further reduced to a variable with the most directly interpretable meaning to minimize the number of variables. Sensitivity analysis using complete-case data showed that the candidate observed variables selected for SEM were largely consistent between the imputed dataset and the original complete-case dataset (35 and 33 variables, respectively). Additionally, the number of farm workers and age of the farm owner were included as candidate observed variables representing the latent variable Capacity.

The appropriate number of factors for each model was determined using parallel analysis, Velicer’s minimum average partial test (20), and the Bayesian Information Criterion (BIC). Exploratory factor analysis was then conducted to ensure that each latent variable adequately represented the corresponding construct. SEM was performed using the “lavaan” package in R. All observed variables were treated as ordinal variables and estimated using diagonally weighted least squares. Model fit was evaluated using the chi-square (*χ^2^*), Tucker-Lewis Index (TLI), robust RMSEA, and standardized root mean square residual (SRMR).

### 2.4 Sociological factors influencing biosecurity implementation

Relationships between farm characteristics and the implementation of biosecurity measures were also examined. Farm characteristics included farm owner’s age, farm management type (family-owned vs. corporate), presence of family employees, herd size (number of sows and fattening pigs), whether CSF-positive wild boars were reported within the prefecture, and the occurrence of CSF outbreaks on the farm.

Biosecurity measure implementation was evaluated across five categories. Because the number of items differed among categories, the total Likert-scale score within each category was divided by the number of items in the category to calculate the mean score, resulting in a maximum score of 5 per category. The scores of the five categories were then summed, with a possible total score of 25. Relationships between categorical farm characteristics and biosecurity implementation were analyzed using the Wilcoxon rank-sum test.

The impact of nudge-related factors on biosecurity practices was evaluated to identify sociological and behavioral factors that contribute to the effective implementation of biosecurity measures. Specifically, relationships between nudge-related scores and biosecurity implementation scores were analyzed using Spearman’s rank correlation coefficient. All analyses were conducted using R statistical software, version 4.3.1 (21).

## 3 Results

### 3.1 Description of questionnaire responses: farm characteristics, biosecurity practices, awareness, and motivation

The valid response rate was 44.2% (228/516). Response rates varied by region, with relatively high participation by farmers in Shikoku (85.0%, 17/20) and Kyushu (77.1%, 54/70), and lower participation rates in Tohoku (27.6%, 16/58) and Kansai (19.0%, 4/21; Table 3). The mean age of farm owners was 51.6 years (range: 24–82 years). Corporate farms accounted for 77.5% of participating farms (79/102), and 69.8% of the farms (139/199) employed family members. Regarding production type, 85.0% were farrow-to-finish farms, 10.6% were fattening farms, and 9.0% were breeding farms. The mean number of sows on breeding and farrow-to-finish farms was 341.2 (median: 180; quartiles: 66–365). The mean number of fattening pigs on farrow-to-finish and fattening farms was 2,200 (median: 1,259; quartiles: 400–2,800). Reports of infected wild boars within the same prefecture were noted by 39.0% of farms (89/228), and 11.8% of farms (27/228) had experienced CSF outbreaks.

**Table 3:** Questionnaire response rate by region (*n* = 228).

Region	Response rate (%)
Hokkaido	47.1 (33/70)
Tohoku	27.6 (16/58)
Kanto	36.8 (14/38)
Chubu	37.7 (55/146)
Kansai	19.0 (4/21)
Chugoku	34.8 (8/23)
Shikoku	85.0 (17/20)
Kyusyu	77.1 (54/70)
Okinawa	38.6 (27/70)
Total	44.2 (228/516)

Mean scores for each category were used to evaluate the status of biosecurity measure implementation. The mean biosecurity practice scores were 3.9 for introduction of pathogens into the hygienic control area by farm staff, 3.7 for introduction of pathogens into pig houses by farm staff, 3.7 for spread of pathogens within pig houses, 4.0 for introduction of pathogens onto the farm by external visitors, and 3.7 for introduction of pathogens into pig houses by wildlife (Supplementary Table 1).

With respect to nudge-related questions (Supplementary Table 2), LHSCs were rated as having the greatest influence on biosecurity measure decision-making (mean score: 4.6), followed by veterinarians (4.4), animal pharmaceutical suppliers (3.7), and neighboring farmers (3.3, *p* < 0.05), as a Messenger. With regard to Incentives, farmers were significantly more strongly motivated to implement biosecurity measures by the desire to minimize the risk of infection (4.5) than by expectations of increased income (3.1, *p* < 0.001). In terms of social Norms, the tendency to maintain a level of biosecurity comparable to that of neighboring farms (3.6) was significantly stronger than concerns over insufficient biosecurity practices at neighboring farms (3.1, *p* < 0.001).

Farmers generally agreed that implementing biosecurity measures should be part of routine farm work (4.0), a Default, and recognized the importance of such measures in daily operations (4.4), a Priming. Most farmers believed they could implement biosecurity measures if their effectiveness was clearly understood (4.3), a Priming. A strong attachment to pigs as an Affect also motivated efforts to reduce disease occurrence (4.6). Measures were moderately guided by goal setting (3.9), as a Commitment, whereas willingness to cooperate with other farms to enhance biosecurity was relatively low (2.6).

### 3.2 SEM based on the KAP-C and PMT frameworks

The distribution of biosecurity scores by category showed mild skewness, and Box-Cox transformation (22) was applied to normalize the data prior to SEM (*λ* = 2.03). Parallel analyses suggested up to five factors, whereas Velicer’s minimum average partial test and the minimum BIC indicated that a single factor was sufficient. Although fewer factors could statistically explain the data, the KAP-C and PMT models are theoretically composed of four and three factors, respectively, making it appropriate to treat them as latent variables in SEM.

**Figure 2:**
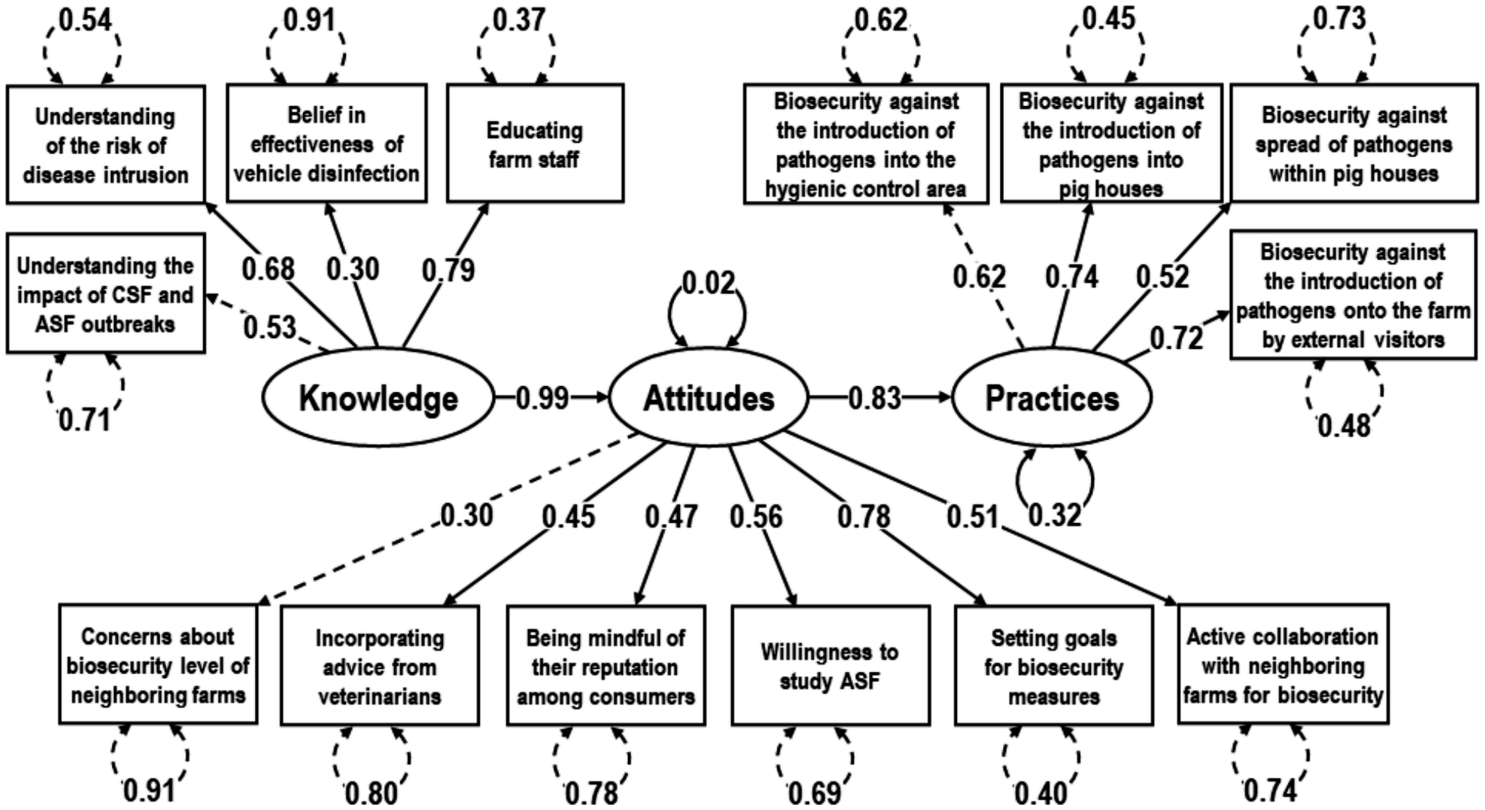
Path diagram of SEM based on KAP (Knowledge-Attitude-Practice). Ellipses and rectangles indicate latent and observed variables, respectively. Values on arrows indicate standardized factor loadings. Curved arrows connected to latent and measured variables indicate disturbances.

In the KAP-C model, Knowledge was significantly associated with Attitude (coefficient = 0.99 [95% confidence interval (CI): 0.91–1.07], *p* < 0.001), and Attitude was positively associated with biosecurity Practices (coefficient = 0.83 [95% CI: 0.74–0.91], *p* < 0.001, Table 4 and Figure 2). Knowledge was characterized by understanding disease risks and staff education. Attitude was characterized by motivation, reputational awareness, and regional collaboration. Practice was significantly associated with four of the five biosecurity practice categories: prevention of pathogen intrusion into hygienic control areas and pig houses, visitor management, and within-farm pathogen control. However, practice to prevent introduction of pathogens into pig houses via wildlife intrusion was not a significant factor. The measurement model for Capacity showed insufficient empirical support, as both observed variables (number of employees and owner age) had non-significant factor loadings (*p* = 0.163 and *p* = 0.260, respectively). In addition, the structure paths from Capacity to Knowledge and Practice were not statistically significant. Therefore, Capacity was not included in the final model. The final KAP model excluding Capacity demonstrated good fit to the data (Table 4: TLI = 1.011, Robust RMSEA = 0.049 and SRMR = 0.053).

**Table 4:** Results of structural equation modeling based on the KAP framework.

Variable	Coefficient (95% confidence interval)	*p* value
Structure		
Knowledge to attitude	0.99 (0.91–1.07)	<0.001
Attitude to practice	0.83 (0.74–0.91)	<0.001
Regression		
Knowledge to		
Understanding the impact of CSF or ASF outbreaks on the region or country	0.53 (0.39–0.68)	<0.001
Implementation of biosecurity measures through understanding the risk of diseases entering	0.68 (0.57–0.78)	<0.001
Belief in the prevention of CSF and ASF intrusion onto the farm by thorough disinfection of vehicles entering the farm	0.30 (0.18–0.43)	<0.001
Educating farm staff on biosecurity practices	0.79 (0.72–0.86)	<0.001
Attitude to		
Concerns about inadequate hygiene management conditions at neighboring pig farms	0.30 (0.17–0.43)	<0.001
Incorporating advice from veterinarians	0.45 (0.31–0.59)	<0.001
Being mindful of one’s reputation with wholesalers and consumers	0.47 (0.36–0.57)	<0.001
Willingness to study about ASF, for which there is potential risk of entry into Japan	0.56 (0.45–0.66)	<0.001
Setting goals for practicing biosecurity	0.78 (0.71–0.84)	<0.001
Active collaboration with other farms to enhance hygiene levels in the region	0.51 (0.41–0.61)	<0.001
Practice to		
Biosecurity against the introduction of pathogens into the hygienic control area (category a)	0.62 (0.50–0.74)	<0.001
Biosecurity against the introduction of pathogens into pig houses (category b)	0.74 (0.64–0.85)	<0.001
Biosecurity against spread of pathogens within pig houses (category c)	0.52 (0.38–0.65)	<0.001
Biosecurity against the introduction of pathogens into the farm by external visitors (category d)	0.72 (0.61–0.83)	<0.001

Fit measures	Value
Number of observations	228
Degrees of freedom	75
*χ*^2^ *p* value	0.956
Tucker-lewis index	1.011
Robust root mean square error of approximation	0.049
Standardized root mean square residual	0.053

**Figure 3:**
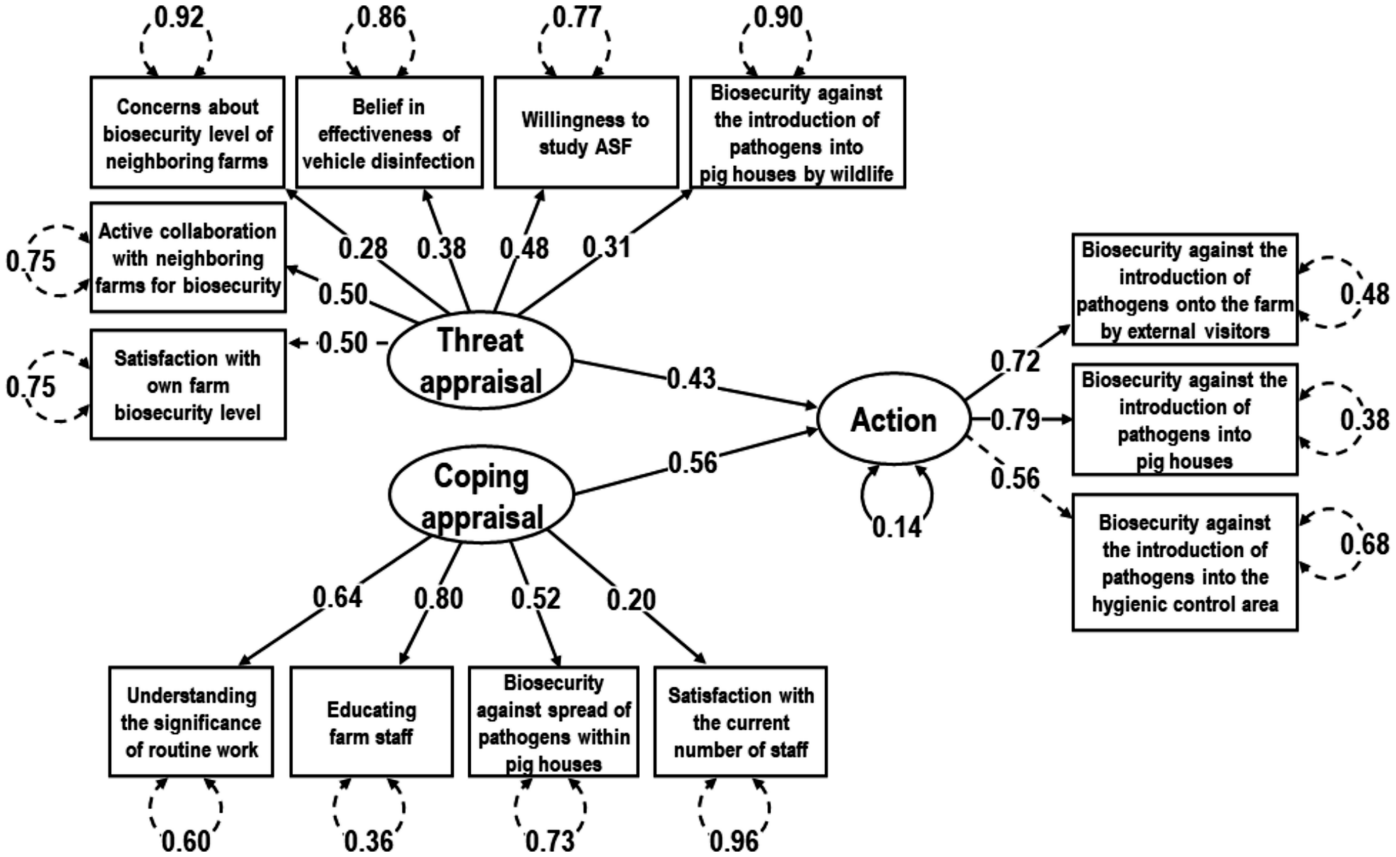
Path diagram of SEM based on the PMT (Protection Motivation Theory). Ellipses and rectangles indicate latent and observed variables, respectively. Values on arrows indicate standardized factor loadings. Curved arrows connected to latent and measured variables indicate disturbances.

In the PMT framework, the model showed acceptable fit to the data (TLI = 0.984, Robust RMSEA = 0.067 and SRMR = 0.05), and higher levels of Threat and Coping appraisals were significantly associated with biosecurity practices, Action (Threat appraisal: coefficient = 0.43 [95% CI: 0.07–0.79], *p* = 0.019; Coping appraisal: 0.56 [95% CI: 0.22–0.90], *p* = 0.001, Table 5 and Figure 3). Threat appraisal was explained by satisfaction with on-farm biosecurity, perceived need for regional measures, motivation to acquire knowledge, and awareness of wildlife intrusion prevention. Coping appraisal was explained by understanding the importance of routine practices, staff education, and farm capacity factors, such as sufficient labor and the ability to implement all-in/all-out systems. Implementation of practices (Action) was primarily driven by measures that prevent pathogen intrusion via vehicles, footwear, and visitors (*p* < 0.01).

**Table 5:** Results of structural equation modeling based on the PMT framework.

Variable	Coefficient (95% confidence interval)	*p* value
Structure		
Threat appraisal to Action	0.43 (0.07–0.79)	0.019
Coping appraisal to Action	0.56 (0.22–0.90)	0.001
Regression		
Threat appraisal to		
Satisfaction with own farm biosecurity measures	0.50 (0.37–0.64)	<0.001
Active collaboration with other farms to enhance hygiene levels in the region	0.50 (0.37–0.63)	<0.001
Concerns about inadequate hygiene management conditions at neighboring pig farms	0.28 (0.14–0.43)	<0.001
Belief in the prevention of CSF and ASF intrusion onto the farm by thorough disinfection of vehicles entering the farm	0.38 (0.25–0.51)	<0.001
Willingness to study ASF due to the potential risk of entry into Japan	0.48 (0.35–0.61)	<0.001
Biosecurity against the introduction of pathogens into pig houses via wildlife	0.31 (0.16–0.46)	<0.001
Coping appraisal to		
Understanding the significance of the biosecurity measures currently performed as part of routine work	0.64 (0.52–0.75)	<0.001
Educating farm staff regarding biosecurity practices	0.80 (0.72–0.88)	<0.001
Biosecurity against the spread of pathogens within pig houses	0.52 (0.39–0.66)	<0.001
Belief that the current number of farm staff is sufficient	0.20 (0.05–0.36)	0.009
Action to		
Biosecurity against the introduction of pathogens into the hygienic control area (category a)	0.56 (0.45–0.68)	<0.001
Biosecurity against the introduction of pathogens into pig houses (category c)	0.79 (0.69–0.89)	<0.001
Biosecurity against the introduction of pathogens onto the farm by external visitors (category d)	0.72 (0.62–0.82)	<0.001

Fit measures	Value
Number of observations	228
Degrees of freedom	62
*χ*^2^ *p* value	0.080
Tucker-Lewis Index	0.984
Robust root mean square error of approximation	0.067
Standardized root mean square residual	0.064

### 3.3 Relationships between farm characteristics and biosecurity implementation

Farm owners under 50 years of age had a significantly higher hygiene score (19.5) compared with those aged 50 years and older (18.5, *p* = 0.050). Regarding business structure, corporate farms had a significantly higher hygiene score (20.1) than family-run farms (*p* < 0.001). Similarly, farms not employing family members had a higher hygiene score (20.1) than farms employing family members (18.8, *p* = 0.009). Across all production types, large-scale farms consistently exhibited higher hygiene scores than small-scale farms (*p* < 0.001).

Farms located in prefectures with reported CSF-infected wild boars had a slightly higher hygiene score (19.6) than farms in prefectures without such reports (18.7, *p* = 0.062). Farms with a history of previous CSF outbreaks had a significantly higher hygiene score (21.3) than farms with no history of outbreaks (18.7, *p* < 0.001).

### 3.4 Relationships between nudge-related scores and biosecurity implementation scores

Distinct patterns were observed in the correlations between nudge-related items and biosecurity implementation scores (Table 6). Among Messenger items, veterinarians (*ρ* = 0.24, *p* < 0.001) and LHSCs (*ρ* = 0.16, *p* = 0.014) were positively correlated with biosecurity implementation score, with the correlation for LHSCs being modest. Within Incentives, biosecurity scores were slightly positively correlated with perceived threat, such as the belief that biosecurity measures can prevent the introduction of CSF/ASF pathogens via wild animal intrusion (*ρ* = 0.17, *p* = 0.010) and vehicle disinfection (*ρ* = 0.21, *p* = 0.001). By contrast, implementing measures to increase revenue showed no correlation (*ρ* = 0.01, *p* = 0.893). For Norms, reputation awareness of veterinarians (*ρ* = 0.26, *p* < 0.001), wholesalers and consumers (*ρ* = 0.24, *p* < 0.001), and LHSCs (*ρ* = 0.18, *p* = 0.006) was positively correlated with biosecurity implementation score, whereas the desire to match biosecurity measures of neighboring farms was not correlated with biosecurity score (*ρ* = 0.02, *p* = 0.789).

**Table 6:** Relationships between nudge-related scores and biosecurity implementation scores.

Item	Correlation coefficient	*p* value
Messenger (key person influencing hygiene improvement)		
Veterinarian	0.24	<0.001
Livestock Hygiene Service Centers (LHSCs)	0.16	0.014
Animal pharmaceutical suppliers	0.01	0.932
Neighboring farmers	0.10	0.118
Incentives (perceived threat and economic benefits)		
Beliefs about preventing CSF/ASF introduction by:		
Preventing wild animal intrusion	0.17	0.010
Vehicle disinfection	0.21	0.001
Hygiene management for people entering the farm	0.13	0.050
Feed and water hygiene	0.09	0.180
Pig house hygiene	0.15	0.026
Concerns about inadequate hygiene management conditions on neighboring pig farms	0.18	0.006
Implementation of biosecurity measures in order to increase revenue	0.01	0.893
Understanding the significance of the biosecurity measures currently performed as part of a routine	0.38	<0.001
Belief that if the effectiveness is clearly evident, biosecurity measures can be implemented	0.15	0.024
Norms (awareness of social evaluation and peer conformity)		
Awareness of reputation among:		
Wholesalers and consumers	0.24	<0.001
Neighboring pig farmers	0.13	0.051
Veterinarians	0.26	<0.001
LHSCs	0.18	0.006
Desire to maintain biosecurity comparable to neighboring farms	0.02	0.789
Defaults (routine and habitual practices)		
Perception that biosecurity measures can be implemented as routine practices	0.19	0.004
Salience (attention to new practices)		
Willingness to accept new practices	0.21	0.001
Priming (trigger by external stimuli)		
Consideration of hand sanitization on the farm due to the impact of COVID-19	0.45	<0.001
Affect (Emotional influence)		
Desire to reduce diseases in pigs out of care and affection	0.12	0.060
Commitments (self-set and cooperative goals)		
Setting goals for practicing biosecurity	0.46	<0.001
Active collaboration with other farms to enhance hygiene level in the region	0.35	<0.001

In Defaults, the perception of implementing biosecurity measures as a routine practice was slightly positively correlated with biosecurity implementation score (*ρ* = 0.19, *p* = 0.004). For Salience, willingness to adopt new practices was positively correlated with biosecurity score (*ρ* = 0.21, *p* = 0.001). Among Priming items, consideration of hand sanitization due to the impact of COVID-19 showed a positive correlation with biosecurity score (*ρ* = 0.45, *p* < 0.001). In Affect, a weak positive trend was noted between the desire to reduce diseases in pigs out of care and affection and the biosecurity implementation score (*ρ* = 0.12, *p* = 0.060). Finally, in Commitments, setting self-defined biosecurity goals (*ρ* = 0.46, *p* < 0.001) and active collaboration with other farms to enhance regional hygiene (*ρ* = 0.35, *p* < 0.001) were positively associated with higher biosecurity scores.

## 4 Discussion

This study was conducted to explore effective approaches for providing biosecurity guidance to pig farmers by elucidating the decision-making structure underlying the implementation of biosecurity measures by farmers and by identifying sociological factors that influence implementation.

Farm characteristics, nudge-related factors, and awareness were associated with the implementation of biosecurity measures. However, although several nudge-related correlations were observed, their effect sizes were relatively modest and should therefore be interpreted with caution when considering intervention design. Among farm characteristics, higher implementation levels were noted on farms with younger owners, corporate management, absence of family employees, and larger scale, reflecting relatively higher capacity. These findings are consistent with previous studies reporting that larger farms tend to implement more biosecurity measures, likely due to greater investment capacity and the presence of successors (8). However, in SEM based on the KAP-C framework, the latent variable Capacity did not show any significant effects to the latent variables Knowledge or Practice, which was a contradictory to previous report (8). Therefore, the framework is hereafter referred to as the KAP. This result suggests that the observed variables used to represent Capacity (i.e., number of employees and owner age) may have been insufficient, or that Capacity may not function as a direct determinant of behavior, but rather as a contextual background factor influencing the development of KAP. Although Capacity was not significant in the SEM analysis, the relationship between farm characteristics and biosecurity implementation in this study suggests that capacity may provide a key foundation for farmers to implement biosecurity using the resources.

On-farm staff education was strongly associated with the latent variable Knowledge in the KAP model and Coping appraisal in the PMT model. In the PMT framework, Coping appraisal could be partially explained by response efficacy, indicating that on-farm education promotes practical implementation by enhancing staff knowledge and understanding of the effectiveness of biosecurity measures. This interpretation is further supported by the high level of agreement among farmers with the statement that they would implement measures if they understood their effectiveness. This result explains the significant relationships between the latent variables in the KAP model well. It is also important to note that overly complex information may cause confusion and reduce motivation to implement biosecurity measures, creating what has been described as ‘harmful sludge’ (23).

Farms with a history of CSF outbreaks also tended to implement biosecurity measures at higher rates. Furthermore, a positive relationship was observed between perceived threat and biosecurity implementation, suggesting that awareness of infection risks motivates protective actions. This relationship was also confirmed in the KAP model through the latent variable knowledge, which had significant relationships with variables indicating understanding risks of ASF and CSF, and in the PMT model through Threat appraisal. By contrast, the implementation of biosecurity measures for profit-seeking purposes in Incentive showed no significant correlation with actual implementation, indicating that incorporating financial incentives in hygiene guidance by LHSCs and veterinarians may provide limited effectiveness in behavior change. These findings appear to partially contradict previous research that emphasized the importance of presenting scientifically grounded cost-effectiveness information (24) and developing models to evaluate biosecurity measures as decision-support tool (25). On the other hand, Incentives in nudge theory have been reported to promote behavior more through the desire to avoid losses than to pursuit of profit (26). Furthermore, in Japan, social norms are known to strongly influence behavior (27), and small-scale or family-run farms are particularly likely to be affected by social factors, including a conservative mindset that favors maintaining the status quo (28). These points suggest that understanding decision-making in biosecurity practices requires considering both economic factors and the social contexts.

Guidance and evaluation from LHSCs and veterinarians were important factor influencing farmers’ decision-making on biosecurity implementation. In the KAP-based SEM, advice from veterinarians remained a significant factor in explaining farmers’ attitudes, which in turn was associated with greater implementation of biosecurity practices. These findings indicate that guidance and feedback from LHSCs and veterinarians can serve as key triggers for enhancing biosecurity, consistent with previous reports (9). Furthermore, views of consumers and wholesalers, a Norm, may also affect biosecurity practice implementation by farmers.

The results of this study indicated that when farmers understand the effectiveness of biosecurity measures, they are more likely to incorporate these measures into their daily routines, which in turn promotes consistent implementation. Moreover, previous reports demonstrated that establishing consistent routines is essential for maintaining high levels of biosecurity (5, 29). In particular, receiving clear, evidence-based information from Messengers such as LHSCs and veterinarians appears to facilitate behavioral changes and provides a practical pathway for integrating biosecurity measures into routine farm practices.

On the other hand, the results of positive correlation between the Likert scales of relying on Messengers such as veterinarians and LHSCs and the implementation level of biosecurity measures suggest the difficulty of approaching farmers who do not listen to the guidance from such Messengers. Unless such farmers receive animal health information, the KAP process would not occur, and high risk of disease introduction would remain in the area. Several countries introduced livestock farm registration scheme accompanied by evaluation of animal health knowledge (30, 31). In Australia, a tool is provided for farmers to create biosecurity plans tailored to their own farms, enabling them to assess risks, prioritized measures, and develop practical hygiene management plans (32). In addition, there still is a room for improving knowledge delivery to farmers. For example, collaboration with feed company or farmers’ association in hygiene guidance may increase the chance of delivering animal health information to farmers.

This study has several limitations. First, in prefectures where CSF outbreaks were reported between September 2018 and October 2019, the survey was conducted among farms with CSF outbreaks and neighboring farms. Therefore, the representativeness of these prefectures is limited. Second, variation in response rates across regions may have introduced non-response bias, as farms with highly biosecurity awareness may have been more likely to participate in the survey, potentially limiting the representativeness of the sample and influencing the analytical results. Third, because this study evaluated the implementation of biosecurity measures using self-reported data collected through a mail survey, responses may have been affected by variation in question interpretation, and self-reported answers may not necessarily accurately reflect actual on-farm biosecurity practices. In contrast, surveys conducted by MAFF evaluate compliance with the Biosecurity Standards though inspections by LHSCs, and reported compliance rate are extremely high (33), which may indicate a potential influence of social desirability bias. In this regard, the combination of a mailed survey and Likert-scale questions used in the present study may be yielded more honest self-reported data. However, future studies should consider validation using farm inspections, veterinary records, and follow-up interviews.

In conclusion, our findings further demonstrate that understanding the effectiveness of interventions directly impacts farmers’ implementation behaviors and offer important insights that could be useful in designing future intervention strategies. Effective messages delivered to farmers may include content that clearly explains effective biosecurity measures, accurately conveys threats of diseases, fosters a sense of care for the pigs among farmers, and encourages engagement in biosecurity practices at the community level. The model of delivery of animal health knowledge to farmers can be improved by enhanced collaboration between LHSCs, veterinarians, and other farming related public service and industries. Future research and practical applications should focus on field interventions using the effective strategies identified in this study.

## Data Availability

The raw data supporting the conclusions of this article will be made available by the authors, without undue reservation.
