# Supplementary material for: Decision-making structure and nudges for Japanese pig farmers to implement biosecurity measures against classical swine fever outbreaks
Source: Front Vet Sci. 2026 Jun 24;13:1865451. doi: 10.3389/fvets.2026.1865451 (PMC13341457; doi:10.3389/fvets.2026.1865451)
Supplement: Supplementary file 1 [file Table_1.DOCX]

Supplementary Material

# Supplementary Tables

**Supplementary Table 1. Mean scores for biosecurity practices.**

| **Biosecurity practices** | **Mean score** |
| --- | --- |
| **a) Introduction of pathogens into the hygienic control area by farm staff** | **3.9** |
| Disinfecting farm staff vehicles | 4.6 |
| Properly adjusting the concentration of disinfectant for vehicles | 3.9 |
| Clarifying the border of the hygienic control area | 4.2 |
| Conducting showering-in | 2.6 |
| Changing into special clothing | 4.1 |
| **b) Introduction of pathogens into pig houses by farm staff** | **3.7** |
| Daily washing and disinfecting of special clothing for each pig house | 4.6 |
| Changing into special clothing at the entrance of each pig house | 2.8 |
| Changing into special boots at the entrance of each pig house | 3.9 |
| Establishing clear zones for where to place outer and inner boots | 3.8 |
| Disinfecting hands or wearing hygienic gloves | 3.7 |
| **c) Spread of pathogens within pig houses** | **3.7** |
| No moving of pigs by having them walk on the ground | 3.8 |
| Adoption of all-in and all-out for pig pens | 3.9 |
| Disinfection of the pens after an all-out | 3.9 |
| Adoption of all-in and all-out for pig rooms | 3.4 |
| Disinfection of the rooms after an all-out | 3.5 |
| **d) Introduction of pathogens into the farm by external visitors** | **4.0** |
| Restricting entry of feed transport vehicle into hygienic control area | 2.1 |
| Disinfecting veterinarians’ vehicles | 4.2 |
| Changing into special boots by veterinarians | 4.7 |
| Cleaning veterinarians’ hands before providing veterinary services | 4.7 |
| Disinfecting construction tools transported onto the farm | 4.2 |
| Changing into special clothing by representatives of repair services | 3.6 |
| Changing into special boots by facility construction services | 4.2 |
| Disinfecting hands or wearing hygienic gloves by facility construction services | 3.7 |
| Restricting entry of visitors not related to the farm | 4.5 |
| **e) Introduction of pathogens into pig houses by wildlife** | **3.7** |
| Preventing wildlife intrusion into areas used to store pig carcasses | 4.4 |
| Preventing the intrusion of mice and other small wild animals into pig houses | 2.6 |
| Preventing the intrusion of cats, raccoons, and other medium-sized wild animals into the hygienic control area | 3.4 |
| Preventing the intrusion of feral cats, raccoons, and other medium-sized wild animals into pig houses | 4.0 |
| Preventing the intrusion of wild birds into pig houses | 3.9 |

**Supplementary Table 2. Mean scores on farmers’ awareness and motivation.**

| **Items** | **Mean score** |
| --- | --- |
| Incorporating advice from veterinarians | 4.4 |
| Incorporating advice from LHSCs | 4.6 |
| Incorporating advice from neighboring pig farmers | 3.3 |
| Incorporating advice from animal pharmaceutical suppliers | 3.7 |
| Understanding the impact of CSF or ASF outbreaks on farms | 4.8 |
| Understanding the impact of CSF or ASF outbreaks on the region or country | 4.6 |
| Concerns about the inadequate hygiene management conditions on neighboring pig farms | 3.1 |
| Belief in the prevention of CSF intrusion into the farm by vaccination against CSF | 2.5 |
| Satisfaction with own farm biosecurity measures | 3.0 |
| Implementation of biosecurity measures in order to increase revenue | 3.1 |
| Implementation of biosecurity measures because of the understanding of the risk of diseases entering the farm | 4.5 |
| Belief in the prevention of CSF and ASF intrusion into the farm by preventing the intrusion of wild animals | 2.9 |
| Belief in the prevention of CSF and ASF intrusion into the farm by thorough disinfection of vehicles entering the farm | 2.9 |
| Belief in the prevention of CSF and ASF intrusion into the farm by hygiene management for individuals entering the farm | 3.0 |
| Belief in the prevention of CSF and ASF intrusion into the farm by hygiene management of feed and water hygiene | 2.9 |
| Belief in the prevention of CSF and ASF intrusion into the farm by hygiene management in the pig houses | 3.1 |
| Being mindful of the one’s reputation by wholesalers and consumers | 4.3 |
| Being mindful of the one’s reputation by the neighboring pig farmers | 3.3 |
| Being mindful of the one’s reputation by veterinarians | 3.9 |
| Being mindful of the one’s reputation by LHSCs | 4.1 |
| Desire to maintain biosecurity at a level similar to that on neighboring pig farms | 3.6 |
| Belief that if the effectiveness is clearly evident, biosecurity measures can be implemented | 4.3 |
| Understanding the significance of the biosecurity measures currently performed as part of a routine | 4.4 |
| Belief that biosecurity measures can be implemented through routine practices | 4.0 |
| Willingness to accept new practices | 2.6 |
| Conducting farm staff education on biosecurity practices | 3.9 |
| Participation in workshops on biosecurity measures for infectious diseases in livestock | 3.3 |
| Conducting a study on farm HACCP and J-GAP | 2.6 |
| Engaging in a study of livestock hygiene information distributed by LHSCs | 3.9 |
| Willingness to study the current CSF epidemic | 3.7 |
| Willingness to study ASF is potentially a risk of entry into Japan | 3.9 |
| Interest in branding that can promote the thorough implementation of biosecurity measures | 3.1 |
| Consideration of hand sanitization on the farm due to the influence of COVID-19 | 3.8 |
| Use of agriculture magazines as a reference for biosecurity practices | 3.5 |
| Desire to reduce diseases in pigs out of care and affection | 4.6 |
| Setting goals for practicing biosecurity | 3.9 |
| Active collaboration with other farms to enhance hygiene level in the region | 2.6 |
| Belief that the current number of farm staff is sufficient | 3.4 |
| Belief that there are enough labor hours to implement the current biosecurity measures | 3.4 |
| Belief that budgets are sufficient to implement the current biosecurity measures | 3.0 |
